# Supplementary material for: Implementation of different HIV self-testing models with implications for HIV testing services during the COVID-19 pandemic: study protocol for secondary data analysis of the STAR Initiative in South Africa
Source: BMJ Open. 2021 May 18;11(5):e048585. doi: 10.1136/bmjopen-2020-048585 (PMC8130734; doi:10.1136/bmjopen-2020-048585)
Supplement: Author's manuscript [file bmjopen-2020-048585.draft_revisions.pdf]

BMJ Open

BMJ Open is committed to open peer review. As part of this commitment we make the peer review history of every article we publish publicly available.

When an article is published we post the peer reviewers' comments and the authors' responses online. We also post the versions of the paper that were used during peer review. These are the versions that the peer review comments apply to.

The versions of the paper that follow are the versions that were submitted during the peer review process. They are not the versions of record or the final published versions. They should not be cited or distributed as the published version of this manuscript.

BMJ Open is an open access journal and the full, final, typeset and author-corrected version of record of the manuscript is available on our site with no access controls, subscription charges or pay-per-view fees (http://bmjopen.bmj.com).

If you have any questions on BMJ Open's open peer review process please email info.bmjopen@bmj.com

BMJ Open

Implementation of Different HIV Self-Testing Models with Implications for HIV testing Services during the COVID-19 Pandemic: Study Protocol for Secondary Data Analysis of STAR South Africa Initiative

Journal:	*BMJ Open*
Manuscript ID	bmjopen-2020-048585
Article Type:	Protocol
Date Submitted by the Author:	02-Jan-2021

Complete List of Authors:	Majam, Mohammed; Ezintsha, a sub-division of Wits Reproductive Health and HIV Institute, University of Witwatersrand<br>Conserve, Donaldson; George Washington University School of Public Health and Health Services,<br>Zishiri, Vincent; Ezintsha, a sub-division of Wits Reproductive Health and HIV Institute, University of Witwatersrand<br>Haile, Zelalem; Ohio University,<br>Tembo, Angela; Ezintsha, a sub-division of Wits Reproductive Health and HIV Institute, University of Witwatersrand<br>Phiri, Jane<br>Hatzold, Karin; Population Services International<br>Johnson, Cheryl; World Health Organization, Department of HIV/AIDS<br>Venter, Francois

Keywords:	HIV & AIDS < INFECTIOUS DISEASES, International health services < HEALTH SERVICES ADMINISTRATION & MANAGEMENT, Health policy < HEALTH SERVICES ADMINISTRATION & MANAGEMENT

SCHOLARONE™
Manuscripts

I, the Submitting Author has the right to grant and does grant on behalf of all authors of the Work (as defined in the below author licence), an exclusive licence and/or a non-exclusive licence for contributions from authors who are: i) UK Crown employees; ii) where BMJ has agreed a CC-BY licence shall apply, and/or iii) in accordance with the terms applicable for US Federal Government officers or employees acting as part of their official duties; on a worldwide, perpetual, irrevocable, royalty-free basis to BMJ Publishing Group Ltd ("BMJ") its licensees and where the relevant Journal is co-owned by BMJ to the co-owners of the Journal, to publish the Work in this journal and any other BMJ products and to exploit all rights, as set out in our licence.

The Submitting Author accepts and understands that any supply made under these terms is made by BMJ to the Submitting Author unless you are acting as an employee on behalf of your employer or a postgraduate student of an affiliated institution which is paying any applicable article publishing charge ("APC") for Open Access articles. Where the Submitting Author wishes to make the Work available on an Open Access basis (and intends to pay the relevant APC), the terms of reuse of such Open Access shall be governed by a Creative Commons licence – details of these licences and which Creative Commons licence will apply to this Work are set out in our licence referred to above.

Other than as permitted in any relevant BMJ Author's Self Archiving Policies, I confirm this Work has not been accepted for publication elsewhere, is not being considered for publication elsewhere and does not duplicate material already published. I confirm all authors consent to publication of this Work and authorise the granting of this licence.

For peer review only

Implementation of Different HIV Self-Testing Models with Implications for HIV testing Services during the COVID-19 Pandemic: Study Protocol for Secondary Data Analysis of STAR South Africa Initiative

Mohammed Majam[1, 2], Donaldson F. Conserve*[3] , Vincent Zishiri[1], Zelalem T. Haile[4], Angela Tembo[1], Jane Phiri[1], Karin Hatzold[5], Cheryl Johnson[6], Francois Venter[1,2]

[1] Ezintsha,Wits Reproductive Health & HIV Institute (WRHI), University of the Witwatersrand, Johannesburg, South Africa
[2] School of Clinical Medicine, Faculty of Health Sciences, University of the Witwatersrand, Johannesburg, South Africa
[3]Department of Prevention and Community Health, Milken Institute School of Public Health. George Washington University, United States
[4]Department of Social Medicine, Ohio University Heritage College of Osteopathic Medicine, Dublin, US
[5]Population Services International/Society for Family Health, South Africa
[6]World Health Organisation, Geneva, Switzerland

*Corresponding author: dxc341@gmail.com

950 New Hampshire Ave
Washington, D.C 20052
Department of Prevention and Community Health
Milken Institute School of Public Health
George Washington University, United States

For peer review only

Abstract

Introduction: HIV self-testing (HIVST) presents a convenient, private, and home-based approach that removes barriers to providing routine HIV testing services. The Self-Testing AfRica (STAR) Initiative aims to scale up HIVST amongst priority and under-tested populations. HIVST has the potential to help maintain testing services during the social distancing restrictions implemented to prevent the spread of coronavirus diseases 2019 (COVID-19). This project evaluates uptake of the HIVST, linkage to confirmatory testing, and treatment for HIV-positive clients for the STAR South Africa site.

Methods and analysis: This secondary data analysis protocol aims to evaluate different HIVST distribution models from a prospective study implemented during November 2017 and December 2020 by Ezintsha, a sub-division of Wits Reproductive Health and HIV Institute (Wits RHI). Routinely collected distribution and self-reported HIVST outcomes data will be de-identified and analyzed. The main outcomes of interest are HIVST uptake and linkage to care and treatment among HIVST users who report a reactive HIVST result. Additionally, we plan to determine sociodemographic factors associated with HIVST uptake and linkage to care and treatment among HIVST users. Descriptive statistics will be used to describe the variables of interest and modified Poisson regression with robust variance estimation will be performed to identify factors associated with HIVST use and linkage to care and treatment among HIVST users who report a react HIVST result. Risk ratios and 95% confidence interval for the risk ratios will be reported.

Ethics and dissemination: Findings of the secondary data analysis will inform future programmatic implementation of HIVST, scaling up by the National Department of Health, South Africa and other partners operating in similar settings and provide alternative HIV testing and results reporting strategies during COVID-19.

Strengths and limitations of this study

1) We use programme data to investigate the uptake of the HIV self-testing services, linkage to confirmatory testing, and treatment for HIV-positive clients in South Africa.
2) The study findings will inform national policy and scale up of HIV self-testing by the National Department of Health in South Africa and other similar settings.
3) Given the impact of COVID-19 on HIV testing services, HIV self-testing is timely and the study can help to develop new HIV testing strategies during the COVID-19 pandemic.
4) There is limited generalizability of the given participation in self-reporting HIV self-test results is voluntary and not randomized.
5) To address this limitation, we aim to follow-up as much as possible all consenting clients with as many as three reminders presented via the different self-reporting platforms.

For peer review only

INTRODUCTION

An estimated 7.02 million South Africans are living with HIV, making the national population level HIV prevalence at 12.7% and 19.1% among those aged 15-49 [1]. As part of the National Strategic Plan (NSP) on HIV, Sexually Transmitted Infections (STIs) and Tuberculosis, 2017-2022, South Africa has put in place various efforts to promote HIV testing services (HTS) in order to stop new HIV infections and prevent AIDS-related deaths [1]. South Africa's 2017-2022 NSP sets a target to reduce the number of new HIV infections to under 100,000 by 2022 [1]. South Africa has seen an improvement in testing rates with an estimated 86% of the HIV infected population between 15-49 years knowing their status. Routine HTS in South Africa largely takes a provider-based approach (7). This approach requires that individuals present at an HIV Testing location either at the health facility, in the community, or in the home. Whilst there have been gains in increasing access to HTS, an HIV testing gap remains largely amongst men and young people due to existing individual and socio-economic barriers to accessing routine HTS amongst these populations. Closing this testing gap through increased access to HIV testing approaches that can provide HTS to these under tested populations will be critical to achieve the UNAIDS "90-90-90" goals. However, there are new barriers to facility-based HTS in South Africa and elsewhere due to the implementation of quarantine, social distancing, and other restrictions to prevent the spread of coronavirus diseases 2019 (COVID-19)[2 3]. HIV self-testing (HIVST) is a relatively new approach that provides an opportunity to reach, test, and diagnose or prevent infection among populations who were previously considered unreachable even during the COVID-19 pandemic due to the ability for people self-test at home or in the facility while practicing social distancing [4-6].

For peer review only - http://bmjopen.bmj.com/site/about/guidelines.xhtml

The World Health Organization (WHO) recommends HIV Self-Testing (HIVST) as an additional approach to providing HIV testing services that could help with closing this testing gap by increasing access and acceptability for HIV testing [7]. HIVST presents a private and convenient and confidential approach to providing HTS that removes some of the barriers to routine HTS by allowing people to collect their sample and receive their results in the privacy of their home without interacting with a healthcare professional [4]. HIVST also has the potential to reduce costs and save time for the health delivery system and to the end user by triaging out the negative patients [4]. HIVST kits are already available for purchase over-the-counter in several countries, including the United States, United Kingdom, France, South Africa and Kenya and have recently been introduced in the public sector in Malawi, Zambia and Zimbabwe [8]. Since December 2016, WHO has provided guidelines to facilitate HIVST inclusion in national policies [4]. National HIV programmes worldwide have been requested to adapt policy and programme documents, including algorithms and training materials, to fully accommodate HIVST [4]. Consequently, the number of countries with supportive HIVST policies has grown rapidly to 77 with at least 38 countries currently implementing HIVST [9]. To support the use of HIVST in South Africa, guidelines for HIVST implementation were issued by the National Department of Health (NDOH) in February 2018.

STAR Initiative South Africa

Despite the efforts to include HIVST in national policies, the market for HIVST was virtually non-existent in South Africa and other low-and middle-income countries (LMICs) prior to the implementation of the HIV Self-Testing Africa (STAR) Initiative [10]. Key challenges to the development of a healthy HIVST market notably included limited evidence on the public health impact and cost-effectiveness of HIVST, uncertain levels of consumer demand and concerns about

For peer review only

potential social harms amongst others [10]. In response, UNITAID funded the STAR Initiative to establish evidence for HIVST safety, acceptability, feasibility and scalability. The STAR initiative seeks to accelerate access to HIVST in LMICs by creating an enabling environment with regards to HIVST policies, generating diverse demand through multiple distribution channels adapted to the needs of priority populations and creating advocacy for additional financing, as well as accelerate market entry for suppliers at affordable and sustainable prices [10]. The STAR Initiative has two phases and the collaborators include WHO, Population Services International (PSI) and a consortium of partners, including London School of Hygiene and Tropical Medicine, Liverpool School of Tropical Medicine, University College London, and Society for Family Health (SFH) and the University of Witwatersrand Reproductive Health and HIV Institute (Wits RHI) in South Africa [10]. Phase I of the STAR Initiative was implemented in Malawi, Zambia and Zimbabwe (2015-2017) and demonstrated that HIVST can be used accurately, is widely accepted when offered through community, health facility-based and partner-delivered distribution models [11 12] and importantly increases uptake of HIV testing among men, including among male partners of pregnant or postpartum women, adolescents and vulnerable and key populations that do not otherwise use conventional testing services [13]. Building on these promising findings, STAR phase II was expanded to three new Southern African countries—South Africa, Swaziland, and Lesotho to introduce and inform HIVST scale up [14 15] by establishing sustainable systems for HIVST delivery, linkage to confirmatory HIV testing, prevention, and treatment services [15]. The new countries included in phase II represent three of the worst HIV epidemics in the world.

Wits RHI was one of the implementing partner of the STAR Initiative in South Africa and implemented the project in Gauteng, North West, Mpumalanga and Limpopo provinces of South Africa. Approximately 1,2 million HIVST kits were distributed primarily targeting testing men,

For peer review only

young people, and key populations. Under the Wits RHI implemented programme, HIVST was offered if recipient did not know their current HIV status or received an HIV negative result in the last HIV test taken > 3 months ago. HIVST was not offered if recipient was below the age of 12 or known HIV positive or if there was identified risk of social harm such as suicidal thoughts, gender-based violence, or coerced testing.

This secondary data analysis protocol aims to evaluate different HIVST distribution models from a prospective HIVST study implemented in South Africa. The main outcomes of interest are HIVST uptake and linkage to care and treatment among HIVST users who reported a reactive HIVST result by examining uptake of HIVST, proportion of HIVST recipients who reported a reactive HIVST result and sought HIV confirmatory testing and entry into care among clients who received HIVST kits via the different distribution approaches implemented under the STAR Initiative in South Africa.

METHODS AND ANALYSIS

Study Design

The study is a secondary analysis of routinely collected programme data from a prospective study aimed at evaluating different HIVST distribution models implemented during November 2017 and December 2020 by Ezintsha, a sub-division of Wits Reproductive Health and HIV Institute (Wits RHI) (see section on Data collection). The different models implemented by Wits RHI, based on the NDOH HIV Self Screening Guidelines (REF) are presented in Table 1. Briefly, the models include community-based, public sector facility, key population, and private sector targeted distribution. Each model has different channels and implementation strategies.

Sampling and sample size

Because this is a secondary data analysis, we are limited to post hoc power analyses. For one sample binomial test, Cohen (1988) describes effect sizes (ES) as follows: 0.05 (small), 0.15

For peer review only

(medium), 0.25 (large) [16]. With a total of 1,058,381 total HIVST kits distributed, we will have >99% power to detect a small effect size (g = .02) in the expected proportion of HIVST use at α = .05. The smallest sample size for by distribution modality is GP practice where 450 HIVST were distributed. We will be able to detect differences as small as 7% with 84% power at α=0.05.

Data collection

During HIVST distribution, recipients' age, gender, HIV testing frequency as well as partner demographics are collected. Recipients are issued a barcode sticker that accompanies the HIVST kits. Duplicate barcodes are attached to the partner's HIVST kit(s) when provided. This barcode number is entered onto the participant distribution data collection form and is used as the unique identifier for recipients and their associated HIVST kits issued. At distribution; HIVST recipients are also informed of the available options for support with testing, linkage to care as well as self-reported follow-up. Consent is sought from recipients for follow-up through one of the four self-reporting platforms offered on the project, being; telephonic, interactive voice response (IVR), Progressive WebApp (PWA) or WhatsApp messaging (business version). The different self-f reporting platforms were offered to provide participants the ability to choose the option that may work best for them and inform which options are included in national HIVST policy to support the scale up of HIVST and help collect HIVST use and results data nationally. A standardised follow-up questionnaire is administered to consenting HIV self-testers' and seeks responses on; Did you use the test? What was the HIV Self-test result? Did you request confirmatory testing? Did you get confirmatory testing? Were you linked to care? Were you initiated on ART? This set of questions is standard across the four follow-up platforms.

For HIVST recipients preferring follow-up by telephone; up to three telephonic calls are conducted by a trained linkage officer at 2, 4 and 6 weeks post-distribution to get responses on the

For peer review only

above described set of questions. IVR is an automated telephony system that interacts through short messages and calls with HIVST recipients to confidentially self-report their results. The first automated reminder SMS message to use and self-report HIVST results is sent within 24 hours following distribution. A second short message is sent on day 3 (SMS 2). If reporting has not been done at these two interactions the IVR system will automatically call the participant on day 7 to self-report results. Recipients also have the option to make an inbound call to the platform for support and self-reporting at any time post distribution.

PWA is a mobile phone-based tool to support participants through self-testing and eventual confirmatory testing. It is accessible as a mobile-site, but is also downloadable to a phone, where the full site contents can be cached for offline browsing. Users that select the web app receive an SMS 24 hours following distribution, which provide them with a free URL link to access the PWA for registration on the application. Once subscribed, participants are able to access website link and self-report their results.

The Chat-bot on a WhatsApp for business platform allows users to send text and voice messages, make voice and video calls, and share images, documents, user locations, and other media. HIVST recipients who select follow-up and linkage support via the WhatsApp platform receive a WhatsApp message 24 hours following distribution to confirm subscription. Once subscribed, participants are able to access the platform which included terms and conditions, assistance through self-testing process and ultimately self-reporting of their results.

We will merge routinely collected programme distribution and HIVST users' self-reported data. The survey responses are captured and linked to HIVST recipient's distribution data via telephone number and barcode onto the project data base. Data captured into project database is

For peer review only

access controlled and is regularly checked for quality, including data reconciliations and a data verification.

Statistical analysis

Data for the secondary analysis will be de-identified. Continuous variables will be expressed as means (±standard deviations) or median and interquartile range as appropriate. Frequencies and proportions will be used to describe categorical variables. Binomial test will be used to determine whether the proportion of HIVST uptake, linkage to care and receipt of treatment among HIVST users equal to a pre-specified proportion based on a recently published South Africa HIVST study[17]. Differences in the proportions of HIVST uptake, linkage to care and receipt of treatment among HIVST users by distribution modality and other sociodemographic characteristics will be assessed using chi-square test or Fisher's exact test, as appropriate. Unadjusted and multivariable adjusted relative risks of HIVST uptake, linkage to care and receipt of treatment among HIVST will be calculated using modified Poisson regression with robust variance estimation. Risk ratios and 95% confidence interval for the risk ratios will be reported. Two-tailed $P<0.05$ will be considered statistically significant. All analyses will be performed using STATA and SAS 9.4 (SAS Institute Inc., Cary, NC).

Handling missing data

Missing data will be handled using multiple imputation, which will ensure that as minimal as possible incomplete data is dropped. We will create numerous data sets containing different estimates of the missing values maintaining key identifying variables such as age, gender, testing frequency, and distribution location as well as distribution model. Imputed data will be pooled into a study data set for analysis.

Patient and public involvement

For peer review only

This study will not involve patients as it is a secondary analysis of routine programme data. HIVST recipients will not be involved in the development of research questions this study. However, findings from preference, usability and acceptability studies from STAR phase 1 will inform designed of distribution approaches in some models. Wits RHI's Adolescent Health Research Group together with local Community Advisory Boards will provide ongoing guidance towards appropriate processes and procedures for implementation HIVST distribution and follow-up particularly amongst adolescents and men for community-based distribution models.

We plan to share the findings from this study through reports and presentations at local and international conferences. In addition, we will submit manuscripts for publication to peer reviewed journals for wider visibility and utilization of the findings.

Beneficiaries and target audiences

The study findings will contribute to the existing and growing body of evidence on the acceptability and efficacy of deploying HIVST and digital self-reporting and linkage to care platforms to reduce mortality and morbidity from HIV among men, key populations and adolescent boys and girls in South Africa. The project is of interest to a range of audiences including academic and non-academic practitioners, community gate keepers for public health programmes targeting men and young people, HIV programme implementers, governments, and public health funding organisations. A description of the primary and secondary outcomes of this study will help to identify distribution models for government and other funders to invest in scaling- up of effective HIVST models. In addition, potential gaps in the implementation approaches and generalised lessons learnt can be drawn and inferred in future HIV programming for similar settings and target populations.

For peer review only

Limitations

Our analysis plan could have limitations as follows. Firstly, limited generalizability of the findings given participation in self-reporting is voluntary and not randomized. We recognize a high proportion of self-reporting a strong determinant of generalizability of the findings therefore we aim to follow-up as far as possible all consenting clients with as many as three reminders/ engagement opportunities presented via the different self-reporting platforms. Secondly, we are prone to validity and reliability of secondary data, particularly where data is incomplete. The project monitoring and evaluation team aims to ensure the completeness of data. In addition, analysis will be done on pooled imputed data sets to ensure that as minimal as possible incomplete data is dropped. Thirdly, we acknowledge that both obtaining and reporting HIV test result opens to social desirability bias and non-response bias on the data we will collect. The implementation provides self-reporting options to HIVST recipients and ensures anonymous reporting of HIVST use and results data unless where identifiers are consented to this is specifically for clients who are self-reporting on site and are traced and tracked for ART initiation. Fourth, while the analyses will provide results on the outcome for the different distribution modalities, the larger focus of the project and results are to provide preliminary evidence to create a supportive environment for the introduction and integration of HIV self-testing in national policies, strategies, plans, and regulations at the country level. Thus, the focus is less about comparison between different distribution modalities and more about piloting different implementation strategies to collect preliminary data to inform national policies, selection, adaptation, and scaling up of different HIVST delivery and linkage models for different populations.

Discussion

For peer review only - http://bmjopen.bmj.com/site/about/guidelines.xhtml

HIVST has been recommended by WHO and several studies have demonstrated the its acceptability, feasibility, and effectiveness [4 7]. However, there is limited evidence from implementation science research to provide guidance on how to scale up HIVST in LMICs for different hard to reach groups. The STAR Initiative aims to fill this gap and the proposed secondary data will be a crucial step in generating the evidence needed to determine preliminary public health outcomes of HIVST in South Africa [10]. In addition, given the restrictions that coronavirus 2019 (COVID-19) has caused on HIV testing services due to lockdown and social distancing requirements [3 18], the findings of this study are crucial to inform the implementation and expansion of HIVST in order to sustain HIV testing in a safe and socially distant manner that can help reduce exposure to severe acute respiratory syndrome coronavirus 2 (SARS-CoV-2). The different platforms for HIVST delivery evaluated in this study support the call for scaling up of HIVST during COVID-19 using a variety of strategies to ensure safe and appropriate delivery of HIVST and ongoing HIV testing services [5]. Beyond the aforementioned implications, the findings will identify which distribution channels work best and should be adopted by the NDOH and included in the national HIVST policy as well as be incorporated in future WHO HIVST implementation guidelines to support national HIVST implementation plans [15].

Ethical considerations

The Wits RHI STAR programme is implemented according to South Africa's national HIV self-screening guidelines. All clients voluntarily receive the HIV self-testing kits and consented to follow-up is sought prior to be individuals participating in the self-reporting surveys. Refusal of HIVST offer or consent to follow-up does not result in loss of access to any existing HTS services.

The follow-up surveys and secondary data analysis is approved by the University of Witwatersrand Human Research Ethics Committee (ethics reference number 180405). The design

For peer review only

and evaluation of the self-report platforms are approved by the University of Witwatersrand Human Research Ethics Committee ethics reference number 171113, and 180708 for IVRS and PWA and WhatsApp for business respectively.

For peer review only

References

1. National Strategic Plan. South African National Strategic Plan on HIV, TB and STIs 2017-2022 2017 [Available from: http://sanac.org.za/2017/10/24/download-the-full-version-of-the-national-strategic-plan-for-hiv-tb-and-stis-2017-2022/ accessed 18 Nov 2017.
2. Jiang H, Zhou Y, Tang W. Maintaining HIV care during the COVID-19 pandemic. *The Lancet HIV* 2020;7(5):e308-e09.
3. Ponticiello M, Mwanga-Amumpaire J, Tushemereirwe P, et al. “Everything is a Mess”: How COVID-19 is Impacting Engagement with HIV Testing Services in Rural Southwestern Uganda. *AIDS and Behavior* 2020:1.
4. WHO. Guidelines On HIV Self-Testing and Partner Notification - Supplement To Consolidated Guidelines On HIV Testing Services, 2016.
5. Mhango M, Chitungo I, Dzinamarira T. COVID-19 Lockdowns: Impact on Facility-Based HIV Testing and the Case for the Scaling Up of Home-Based Testing Services in Sub-Saharan Africa. *AIDS and Behavior* 2020:1.
6. Dovel K, Shaba F, Offorjebe OA, et al. Effect of facility-based HIV self-testing on uptake of testing among outpatients in Malawi: a cluster-randomised trial. *The Lancet Global Health* 2020;8(2):e276-e87.
7. Organization WH. WHO recommends HIV self-testing: evidence update and considerations for success: policy brief. WHO recommends HIV self-testing: evidence update and considerations for success: policy brief2019.
8. HIV Self-Testing AfRica (STAR) Initiative - Research London school of Hygiene and Tropical Medicine; 2018 [Available from: http://hivstar.lshtm.ac.uk/.
9. UNAIDS. Global AIDS update 2019—communities at the centre: UNAIDS Geneva, 2019.
10. Ingold H, Mwerinde O, Ross AL, et al. The Self-Testing AfRica (STAR) Initiative: accelerating global access and scale-up of HIV self-testing. *Journal of the International AIDS Society* 2019;22:e25249.
11. d’Elbée M, Indravudh PP, Mwenge L, et al. Preferences for linkage to HIV care services following a reactive self-test: discrete choice experiments in Malawi and Zambia. *Aids* 2018;32(14):2043-49.
12. Indravudh PP, Sibanda EL, d’Elbée M, et al. ‘I will choose when to test, where I want to test’: investigating young people's preferences for HIV self-testing in Malawi and Zimbabwe. *Aids* 2017;31:S203-S12.
13. Indravudh PP, Choko AT, Corbett EL. Scaling up HIV self-testing in sub-Saharan Africa: a review of technology, policy and evidence. *Curr Opin Infect Dis* 2018;31(1):14-24. doi: 10.1097/QCO.0000000000000426
14. UNITAID. World’s largest HIV Self-Testing Initiative expands in critical new phase. : World Health Organisation (UNITAID), 2018.
15. Neuman M, Indravudh P, Chilongosi R, et al. The effectiveness and cost-effectiveness of community-based lay distribution of HIV self-tests in increasing uptake of HIV testing among adults in rural Malawi and rural and peri-urban Zambia: protocol for STAR (self-testing for Africa) cluster randomized evaluations. *BMC public health* 2018;18(1):1234.
16. Cohen J. Statistical power analysis for the behavioral sciences: Academic press 2013.
17. Shapiro AE, van Heerden A, Krows M, et al. An implementation study of oral and blood-based HIV self-testing and linkage to care among men in rural and peri-urban KwaZulu-Natal, South Africa. *Journal of the International AIDS Society* 2020;23:e25514.
18. Lagat H, Sharma M, Kariithi E, et al. Impact of the COVID-19 Pandemic on HIV Testing and Assisted Partner Notification Services, Western Kenya. *AIDS and Behavior* 2020:1.

For peer review only

For peer review only

Author's contributions

MM and FV designed the program implementation strategies, the operational study design. DFC and VZ wrote the first draft of the manuscript MM, AT, VZ, MS, MN oversaw the implementation of the program and drafted the standard operating procedures. JP oversaw data management processes. KH provided an oversight of the program implementation. DFC and ZTH helped to revise the manuscript and address major reviewers' comments. All authors took part in the revision, reading, and approved the final version of the protocol manuscript.

Funding

This work was funded by the UNITAID grant 2017-17-SFH-STAR and Bill & Melinda Gates Foundation OPP1132929, the National Institute of Health (R00MH110343: PI, DFC) and the HIV Dissemination Science Training Program for Underrepresented Investigators grant (R25MH080665). The findings and conclusions in this report are those of the authors and do not necessarily represent the views of the funder.

Competing interests

Authors have no competing interests.

For peer review only

Table 1: HIVST distribution models implemented by WitsRHI / Ezintsha

Model	Modality	Description
**Community Based Distribution**	Hotspots	HIVSS was offered to high-risk populations at hotspots through fixed pop-up sites. Hotspots include high foot traffic areas such as busy walkways or shopping centers. After a peer educator provided a demonstration on how to use HIVSS kits, clients were provided with a choice to take a kit home or use it onsite either on their own or with the assistance of the distributor. Clients opting to test onsite were given an opportunity to confirm reactive results. All women and males who indicated having a male partner were offered an HIVSS test kit for their partners. 5% of consenting primary recipients of HIVSS were telephonically followed up for outcomes of HIVSS use, result, linkage to care and uptake of ART. From November 2019, all consenting clients were offered a choice to self-report their outcomes using one of the 3 m-health platforms, specifically, WebApp, WhatsApp or on the interactive Voice Response system (IVRS).
	Transport Hub	The description of this modality is the same as the Community based- Hotspots modality, with a specific focus on distribution in transport hubs including Taxi ranks and Train stations
	Door to Door	Within a specific mapped community or geographic location, peer educators and/or trained counsellors move from one household to another and offer HIVSS to eligible clients residing there. Clients were offered HIVSS with the option of either directly assisted (in presence of health care provider) or unassisted. Clients who self-report reactive results were offered the option to confirm their results onsite. All women and men who had male partners were offered oral HIVSS tests for their partners. To collect information on outcomes of HIVSS use, result, linkage to care and uptake of ART, Peer educators went back to the residences of all consenting clients
	Integrated HTS (mobile)	This modality involved the Integration of HIVSS into existing mobile HIV testing activities. Clients were offered a choice between HIVSS onsite or Rapid Diagnostic Testing (RDT) with a counsellor. HIVSS kits were distributed to individual clients after a demonstration of how to use them. Clients that opted for HIVSS, were only offered onsite testing with the option to confirm a reactive HIVSS result immediately. Clients who confirmed reactive were referred to nearby health facilities for ART initiation. To collect information on outcomes of linkage to care and uptake of ART, linkage officers checked ART registers of referral health facilities. Integration of HIVSS into mobile HTS services did not displace traditional blood based RDT uptake but provided a screening option increased yield and efficiency for triaging clients for further managed health care.

Model	Modality	Description
**Key Population Distribution**	Sex Worker	This modality was implemented alongside the WitsRHI sex worker program, which provides various health services to sex workers at static sex worker clinics and at outreach sites. Sex workers were offered up to 5 HIVSS kits to take for their network which would either be; their non-client sexual partners; their clients; other sex workers not accessing HIV testing services; or member of family or friend considered by the sex worker to be at high risk of HIV. This modality was intended to be a secondary only distribution modality; however, sex workers could use one kit for themselves if they needed to test themselves together with one of their recipients. All consenting sex workers were telephonically followed up to determine the usage, linkage to care and ART initiation status of their networks.
**Public Sector Facility Distribution**	Outpatient Department (OPD)	Within the hospital out-patient waiting areas, peer educators created awareness and provided information on both traditional RDT and HIVSS. Clients were then offered the choice between testing themselves onsite using either a blood or oral based HIVSS test inside a provided cubicle or have RDT with a counsellor. HIVSS Reactive clients were provided with the opportunity to confirm their results and be initiated on ART immediately. Offering HIVSS alongside traditional rapid testing in the out-patient departments not only provides clients with a choice but also a triage system whereby testers who screen non-reactive using HIVSS may exit, allowing counsellors and nurses offering rapid testing enough time to focus on likely reactive testers.
**Private Sector Facility Distribution**	GP Practice & Nurse-led clinics	Clients attending health services at GP practices or Nurse-led Unjani clinics were offered HIV testing using HIVSS during their consultation. After a demonstration was provided by the consulting clinician, clients are offered a private testing space within the facility to test themselves and were asked to self-report their result. Confirmatory testing and ART initiation were available onsite. All clients who self-report a reactive result were offered an HIVSS test to take home for their partners.
**Private Sector Distribution**	Pharmacy	Pharmacists and pharmacist assistants at private pharmacies offered HIVSS to adult clients, 17 and older seeking services that might suggest HIV risk, including emergency contraceptive, treatment for STI's, condoms, lubricants, sexual performance enhancers etc. After a demonstration is offered, clients can choose to either test onsite on their own or take the kit home. Clients who chose to test onsite were given an opportunity to self-report their result to the Pharmacist or pharmacist assistant. All who self-report a reactive result, were offered onsite confirmatory testing when available. All women and men who have male partners were offered oral HIVSS tests for their partners. clients were offered m-health platforms to self-report on use, result, and linkage to care.

For peer review only

Model	Modality	Description
	Workplace	This involved distribution of HIVSS in formal workplaces with a focus on mining, manufacturing, construction, petroleum, agriculture, and security sectors. Employees were offered an HIVSS test kit to take home. All female employees were offered an HIVSS kit for their male partners. A 5% sample of primary consenting recipients of HIVSS were telephonically followed up to obtain information on HIVSS usage, result, linkage to care and ART initiation. Beginning August 2019, clients were also offered m-health platforms to self-report on use, result, and linkage to care.
**Secondary Based Distribution**	Antenatal	After a demonstration and social harm assessment, all 1st visit antenatal attendees were offered HIVSS to take home to support testing their sexual partners. The HIVSS modality was not intended to replace facility based RDT testing but support testing of male partners. Women could take HIVSS kit for herself to support home disclosure. Consenting women were followed up by telephone or at next visit to acquire information on whether HIVSS had been used by partner(s) and for test result and linkage data.
	Index Testing	HIVSS is offered to newly diagnosed HIV positive clients (ART clinic) to facilitate partner notification. Telephonic follow up of HIV positive index to ascertain index partner uptake of HIVSS, linkage of positive sexual partner to confirmative testing and treatment initiation

For peer review only

BMJ Open

Implementation of Different HIV Self-Testing Models with Implications for HIV testing Services during the COVID-19 Pandemic: Study Protocol for Secondary Data Analysis of STAR South Africa Initiative

Journal:	*BMJ Open*
Manuscript ID	bmjopen-2020-048585.R1
Article Type:	Protocol
Date Submitted by the Author:	24-Mar-2021
Complete List of Authors:	Majam, Mohammed; Ezintsha, a sub-division of Wits Reproductive Health and HIV Institute, University of Witwatersrand<br>Conserve, Donaldson; George Washington University School of Public Health and Health Services,<br>Zishiri, Vincent; Ezintsha, a sub-division of Wits Reproductive Health and HIV Institute, University of Witwatersrand<br>Haile, Zelalem; Ohio University,<br>Tembo, Angela; Ezintsha, a sub-division of Wits Reproductive Health and HIV Institute, University of Witwatersrand<br>Phiri, Jane<br>Hatzold, Karin; Population Services International<br>Johnson, Cheryl; World Health Organization, Department of HIV/AIDS<br>Venter, Francois
**Primary Subject Heading**:	HIV/AIDS
Secondary Subject Heading:	Health services research
Keywords:	HIV & AIDS < INFECTIOUS DISEASES, International health services < HEALTH SERVICES ADMINISTRATION & MANAGEMENT, Health policy < HEALTH SERVICES ADMINISTRATION & MANAGEMENT

SCHOLARONE™
Manuscripts

I, the Submitting Author has the right to grant and does grant on behalf of all authors of the Work (as defined in the below author licence), an exclusive licence and/or a non-exclusive licence for contributions from authors who are: i) UK Crown employees; ii) where BMJ has agreed a CC-BY licence shall apply, and/or iii) in accordance with the terms applicable for US Federal Government officers or employees acting as part of their official duties; on a worldwide, perpetual, irrevocable, royalty-free basis to BMJ Publishing Group Ltd ("BMJ") its licensees and where the relevant Journal is co-owned by BMJ to the co-owners of the Journal, to publish the Work in this journal and any other BMJ products and to exploit all rights, as set out in our licence.

The Submitting Author accepts and understands that any supply made under these terms is made by BMJ to the Submitting Author unless you are acting as an employee on behalf of your employer or a postgraduate student of an affiliated institution which is paying any applicable article publishing charge ("APC") for Open Access articles. Where the Submitting Author wishes to make the Work available on an Open Access basis (and intends to pay the relevant APC), the terms of reuse of such Open Access shall be governed by a Creative Commons licence – details of these licences and which Creative Commons licence will apply to this Work are set out in our licence referred to above.

Other than as permitted in any relevant BMJ Author's Self Archiving Policies, I confirm this Work has not been accepted for publication elsewhere, is not being considered for publication elsewhere and does not duplicate material already published. I confirm all authors consent to publication of this Work and authorise the granting of this licence.

For peer review only

Implementation of Different HIV Self-Testing Models with Implications for HIV testing Services during the COVID-19 Pandemic: Study Protocol for Secondary Data Analysis of STAR South Africa Initiative

Mohammed Majam[1, 2], Donaldson F. Conserve*[3] , Vincent Zishiri[1], Zelalem T. Haile[4], Angela Tembo[1], Jane Phiri[1], Karin Hatzold[5], Cheryl Johnson[6], Francois Venter[1,2]

[1] Ezintsha,Wits Reproductive Health & HIV Institute (WRHI), University of the Witwatersrand, Johannesburg, South Africa
[2] School of Clinical Medicine, Faculty of Health Sciences, University of the Witwatersrand, Johannesburg, South Africa
[3]Department of Prevention and Community Health, Milken Institute School of Public Health. George Washington University, United States
[4]Department of Social Medicine, Ohio University Heritage College of Osteopathic Medicine, Dublin, US
[5]Population Services International/Society for Family Health, South Africa
[6]World Health Organisation, Geneva, Switzerland

*Corresponding author: dxc341@gmail.com

950 New Hampshire Ave
Washington, D.C 20052
Department of Prevention and Community Health
Milken Institute School of Public Health
George Washington University, United States

For peer review only

Abstract

Introduction: HIV self-testing (HIVST) presents a convenient, private, and home-based approach that removes barriers to providing routine HIV testing services. The Self-Testing AfRica (STAR) Initiative aims to scale up HIVST amongst priority and under-tested populations. HIVST has the potential to help maintain testing services during the social distancing restrictions implemented to prevent the spread of coronavirus diseases 2019 (COVID-19). This project evaluates linkage to confirmatory testing, and treatment for HIV-positive clients for the STAR South Africa site.

Methods and analysis: This secondary data analysis protocol aims to evaluate different HIVST distribution models from a prospective study implemented during November 2017 and December 2020 by Ezintsha, a sub-division of Wits Reproductive Health and HIV Institute (Wits RHI). Routinely collected distribution and self-reported HIVST outcomes data will be de-identified and analyzed. The main outcomes of interest are linkage to care and treatment among HIVST users who report a reactive HIVST result. Additionally, we plan to determine sociodemographic factors associated with linkage to care and treatment among HIVST users. Descriptive statistics will be used to describe the variables of interest and modified Poisson regression with robust variance estimation will be performed to identify factors associated with linkage to care and treatment among HIVST users who report a react HIVST result. Risk ratios and 95% confidence interval for the risk ratios will be reported.

Ethics and dissemination: The study protocol has been approved by the University of Witwatersrand Human Research Ethics Committee. The dissemination plan for the study findings will include presentations to local, and international health authorities, international conferences, and publications in open access journals.

For peer review only

Strengths and limitations of this study

1) We investigate linkage to confirmatory testing, and treatment for self-testers who report a positive self-test result.
2) We will conduct secondary analysis of data from a prospective study that included different HIVST distribution models.
3) There is limited generalizability of the given participation in self-reporting HIV self-test results is voluntary and not randomized.
4) To address this limitation, we followed up with as many participants as possible via the different self-reporting platforms.

For peer review only

INTRODUCTION

An estimated 7.02 million South Africans are living with HIV, making the national population level HIV prevalence at 12.7% and 19.1% among those aged 15-49 [1]. As part of the National Strategic Plan (NSP) on HIV, Sexually Transmitted Infections (STIs) and Tuberculosis, 2017-2022, South Africa has put in place various efforts to promote HIV testing services (HTS) in order to stop new HIV infections and prevent AIDS-related deaths [1]. South Africa's 2017-2022 NSP sets a target to reduce the number of new HIV infections to under 100,000 by 2022 [1]. South Africa has seen an improvement in testing rates with an estimated 86% of the HIV infected population between 15-49 years knowing their status. Routine HTS in South Africa largely takes a provider-based approach (7). This approach requires that individuals present at an HIV Testing location either at the health facility, in the community, or in the home. Whilst there have been gains in increasing access to HTS, an HIV testing gap remains largely amongst men and young people due to existing individual and socio-economic barriers to accessing routine HTS amongst these populations. Closing this testing gap through increased access to HIV testing approaches that can provide HTS to these under tested populations will be critical to achieve the UNAIDS "90-90-90" goals. However, there are new barriers to facility-based HTS in South Africa and elsewhere due to the implementation of quarantine, social distancing, and other restrictions to prevent the spread of coronavirus diseases 2019 (COVID-19)[2 3]. HIV self-testing (HIVST) is a relatively new approach that provides an opportunity to reach, test, and diagnose or prevent infection among populations who were previously considered unreachable even during the COVID-19 pandemic due to the ability for people self-test at home or in the facility while practicing social distancing [4-6].

For peer review only

The World Health Organization (WHO) recommends HIV Self-Testing (HIVST) as an additional approach to providing HIV testing services that could help with closing this testing gap by increasing access and acceptability for HIV testing [7]. HIVST presents a private and convenient and confidential approach to providing HTS that removes some of the barriers to routine HTS by allowing people to collect their sample and receive their results in the privacy of their home without interacting with a healthcare professional [4]. HIVST also has the potential to reduce costs and save time for the health delivery system and to the end user by triaging out the negative patients [4]. HIVST kits are already available for purchase over-the-counter in several countries, including the United States, United Kingdom, France, South Africa and Kenya and have recently been introduced in the public sector in Malawi, Zambia and Zimbabwe [8]. Since December 2016, WHO has provided guidelines to facilitate HIVST inclusion in national policies [4]. National HIV programmes worldwide have been requested to adapt policy and programme documents, including algorithms and training materials, to fully accommodate HIVST [4]. Consequently, the number of countries with supportive HIVST policies has grown rapidly to 77 with at least 38 countries currently implementing HIVST [9]. To support the use of HIVST in South Africa, guidelines for HIVST implementation were issued by the National Department of Health (NDOH) in February 2018.

STAR Initiative South Africa

Despite the efforts to include HIVST in national policies, the market for HIVST was virtually non-existent in South Africa and other low-and middle-income countries (LMICs) prior to the implementation of the HIV Self-Testing Africa (STAR) Initiative [10]. Key challenges to the development of a healthy HIVST market notably included limited evidence on the public health impact and cost-effectiveness of HIVST, uncertain levels of consumer demand and concerns about

For peer review only

potential social harms amongst others [10]. In response, UNITAID funded the STAR Initiative to establish evidence for HIVST safety, acceptability, feasibility and scalability. The STAR initiative seeks to accelerate access to HIVST in LMICs by creating an enabling environment with regards to HIVST policies, generating diverse demand through multiple distribution channels adapted to the needs of priority populations and creating advocacy for additional financing, as well as accelerate market entry for suppliers at affordable and sustainable prices [10]. The STAR Initiative has two phases and the collaborators include WHO, Population Services International (PSI) and a consortium of partners, including London School of Hygiene and Tropical Medicine, Liverpool School of Tropical Medicine, University College London, and Society for Family Health (SFH) and the University of Witwatersrand Reproductive Health and HIV Institute (Wits RHI) in South Africa [10]. Phase I of the STAR Initiative was implemented in Malawi, Zambia and Zimbabwe (2015-2017) and demonstrated that HIVST can be used accurately, is widely accepted when offered through community, health facility-based and partner-delivered distribution models [11 12] and importantly increases uptake of HIV testing among men, including among male partners of pregnant or postpartum women, adolescents and vulnerable and key populations that do not otherwise use conventional testing services [13]. Building on these promising findings, STAR phase II was expanded to three new Southern African countries—South Africa, Swaziland, and Lesotho to introduce and inform HIVST scale up [14 15] by establishing sustainable systems for HIVST delivery, linkage to confirmatory HIV testing, prevention, and treatment services [15]. The new countries included in phase II represent three of the worst HIV epidemics in the world.

Wits RHI was one of the implementing partner of the STAR Initiative in South Africa and implemented the project in Gauteng, North West, Mpumalanga and Limpopo provinces of South Africa. Approximately 1,2 million HIVST kits were distributed primarily targeting testing men,

For peer review only

young people, and key populations. Under the Wits RHI implemented programme, HIVST was offered if recipient did not know their current HIV status or received an HIV negative result in the last HIV test taken > 3 months ago. HIVST was not offered if recipient was below the age of 12 or known HIV positive or if there was identified risk of social harm such as suicidal thoughts, gender-based violence, or coerced testing.

This secondary data analysis protocol aims to evaluate different HIVST distribution models from a prospective HIVST study implemented in South Africa. The main outcomes of interest are linkage to care and treatment among HIVST users who reported a reactive HIVST result by examining proportion of HIVST recipients who reported a reactive HIVST result and sought HIV confirmatory testing and entry into care among clients who received HIVST kits via the different distribution approaches implemented under the STAR Initiative in South Africa.

METHODS AND ANALYSIS

Study Design

The study is a secondary analysis of routinely collected programme data from a prospective study aimed at evaluating different HIVST distribution models implemented during November 2017 and December 2020 by Ezintsha, a sub-division of Wits Reproductive Health and HIV Institute (Wits RHI) (see section on Data collection). The different models implemented by Wits RHI were based on the NDOH HIV Self Screening Guidelines are presented in Table 1. Briefly, the models included community-based, public sector facility, key population, and private sector targeted distribution. Each model had different channels and implementation strategies.

Data collection

During HIVST distribution, recipients' age, gender, HIV testing frequency as well as partner demographics were collected. Recipients were issued a barcode sticker that accompanies

For peer review only

the HIVST kits. Duplicate barcodes were attached to the partner's HIVST kit(s) when provided. This barcode number was entered onto the participant distribution data collection form and was used as the unique identifier for recipients and their associated HIVST kits issued. At distribution, HIVST recipients were also informed of the available options for support with testing, linkage to care as well as self-reported follow-up. Consent was sought from recipients for follow-up through one of the four self-reporting platforms offered on the project: telephonic, interactive voice response (IVR), Progressive WebApp (PWA) or WhatsApp messaging (business version). The different self- reporting platforms were offered to provide participants the ability to choose the option that may work best for them and inform which options will be included in national HIVST policy to support the scale up of HIVST and help collect HIVST use and results data nationally. A standardised follow-up questionnaire was administered to consenting HIV self-testers' and collected responses on; Did you use the test? What was the HIV Self-test result? Did you request confirmatory testing? Did you get confirmatory testing? Were you linked to care? Were you initiated on ART? This set of questions was standard across the four follow-up platforms.

For HIVST recipients preferring follow-up by telephone, up to three telephonic calls were conducted by a trained linkage officer at 2, 4- and 6-weeks post-distribution to get responses on the above described set of questions. IVR is an automated telephony system that interacts through short messages and calls with HIVST recipients to confidentially self-report their results. The first automated reminder SMS message to use and self-report HIVST results was sent within 24 hours following distribution. A second short message was sent on day 3 (SMS 2). If reporting was not been done at these two interactions the IVR system automatically called the participant on day 7 to self-report results. Recipients also had the option to make an inbound call to the platform for support and self-reporting at any time post distribution.

For peer review only

PWA is a mobile phone-based tool to support participants through self-testing and eventual confirmatory testing. It is accessible as a mobile-site, but is also downloadable to a phone, where the full site contents can be cached for offline browsing. Users that selected the web app received an SMS 24 hours following distribution, which provided them with a free URL link to access the PWA for registration on the application. Once subscribed, participants were able to access website link and self-report their results.

The Chat-bot on a WhatsApp for business platform allowed users to send text and voice messages, make voice and video calls, and share images, documents, user locations, and other media. HIVST recipients who selected follow-up and linkage support via the WhatsApp platform received a WhatsApp message 24 hours following distribution to confirm subscription. Once subscribed, participants were able to access the platform which included terms and conditions, assistance through self-testing process and ultimately self-reporting of their results.

We will merge routinely collected programme distribution and HIVST users' self-reported data. The survey responses were captured and linked to HIVST recipient's distribution data via telephone number and barcode onto the project data base. Data captured into project database is access controlled and is regularly checked for quality, including data reconciliations and a data verification.

Statistical analysis

Data for the secondary analysis will be de-identified and the analysis will be conducted between April and July 2021. Continuous variables will be expressed as means (±standard deviations) or median and interquartile range as appropriate. Frequencies and proportions will be used to describe categorical variables. Binomial test will be used to determine whether the proportion of linkage to care and receipt of treatment among HIVST users equal to a pre-specified

For peer review only

proportion based on a recently published South Africa HIVST study[16]. Differences in the proportions of linkage to care and receipt of treatment among HIVST users by distribution modality and other sociodemographic characteristics will be assessed using chi-square test or Fisher's exact test, as appropriate. Unadjusted and multivariable adjusted relative risks of linkage to care and receipt of treatment among HIVST will be calculated using modified Poisson regression with robust variance estimation. Risk ratios and 95% confidence interval for the risk ratios will be reported. Two-tailed $P<0.05$ will be considered statistically significant. All analyses will be performed using STATA and SAS 9.4 (SAS Institute Inc., Cary, NC).

Handling missing data

Missing data will be handled using multiple imputation, which will ensure that as minimal as possible incomplete data is dropped. We will create numerous data sets containing different estimates of the missing values maintaining key identifying variables such as age, gender, testing frequency, and distribution location as well as distribution model. Imputed data will be pooled into a study data set for analysis.

Patient and public involvement

This study will not involve patients as it is a secondary analysis of routine programme data. HIVST recipients will not be involved in the development of research questions this study. However, findings from preference, usability and acceptability studies from STAR phase 1 will inform designed of distribution approaches in some models. Wits RHI's Adolescent Health Research Group together with local Community Advisory Boards will provide ongoing guidance towards appropriate processes and procedures for implementation HIVST distribution and follow-up particularly amongst adolescents and men for community-based distribution models. We plan to share the findings from this study through reports and presentations at local and international

For peer review only

conferences. In addition, we will submit manuscripts for publication to peer reviewed journals for wider visibility and utilization of the findings.

Beneficiaries and target audiences

The study findings will contribute to the existing and growing body of evidence on the acceptability and efficacy of deploying HIVST and digital self-reporting and linkage to care platforms to reduce mortality and morbidity from HIV among men, key populations and adolescent boys and girls in South Africa. The project is of interest to a range of audiences including academic and non-academic practitioners, community gate keepers for public health programmes targeting men and young people, HIV programme implementers, governments, and public health funding organisations. A description of the primary and secondary outcomes of this study will help to identify distribution models for government and other funders to invest in scaling- up of effective HIVST models. In addition, potential gaps in the implementation approaches and generalised lessons learnt can be drawn and inferred in future HIV programming for similar settings and target populations.

Limitations

Our analysis plan could have limitations as follows. Firstly, limited generalizability of the findings given participation in self-reporting is voluntary and not randomized. We recognize a high proportion of self-reporting a strong determinant of generalizability of the findings therefore we aim to follow-up as far as possible all consenting clients with as many as three reminders/ engagement opportunities presented via the different self-reporting platforms. Secondly, we are prone to validity and reliability of secondary data, particularly where data is incomplete. The project monitoring and evaluation team aims to ensure the completeness of data. In addition, analysis will be conducted on pooled imputed data sets to ensure that as minimal as possible

For peer review only

incomplete data is dropped. Thirdly, we acknowledge that both obtaining and reporting HIV test result opens to social desirability bias and non-response bias on the data we will collect. The implementation provides self-reporting options to HIVST recipients and ensures anonymous reporting of HIVST use and results data unless where identifiers are consented to this is specifically for clients who are self-reporting on site and are traced and tracked for ART initiation. Fourth, while the analyses will provide results on the outcome for the different distribution modalities, the larger focus of the project and results are to provide preliminary evidence to create a supportive environment for the introduction and integration of HIV self-testing in national policies, strategies, plans, and regulations at the country level. Thus, the focus is less about comparison between different distribution modalities and more about piloting different implementation strategies to collect preliminary data to inform national policies, selection, adaptation, and scaling up of different HIVST delivery and linkage models for different populations.

Discussion

HIVST has been recommended by WHO and several studies have demonstrated the its acceptability, feasibility, and effectiveness [4 7]. However, there is limited evidence from implementation science research to provide guidance on how to scale up HIVST in LMICs for different hard to reach groups. The STAR Initiative aims to fill this gap and the proposed secondary data will be a crucial step in generating the evidence needed to determine preliminary public health outcomes of HIVST in South Africa [10]. In addition, given the restrictions that coronavirus 2019 (COVID-19) has caused on HIV testing services due to lockdown and social distancing requirements [3 17], the findings of this study are crucial to inform the implementation and expansion of HIVST in order to sustain HIV testing in a safe and socially distant manner that

For peer review only

can help reduce exposure to severe acute respiratory syndrome coronavirus 2 (SARS-CoV-2). The different platforms for HIVST delivery evaluated in this study support the call for scaling up of HIVST during COVID-19 using a variety of strategies to ensure safe and appropriate delivery of HIVST and ongoing HIV testing services [5]. Beyond the aforementioned implications, the findings will identify which distribution channels work best and should be adopted by the NDOH and included in the national HIVST policy as well as be incorporated in future WHO HIVST implementation guidelines to support national HIVST implementation plans [15].

Ethics and dissemination

The Wits RHI STAR programme is implemented according to South Africa's national HIV self-screening guidelines. All clients voluntarily receive the HIV self-testing kits and consented to follow-up is sought prior to be individuals participating in the self-reporting surveys. Refusal of HIVST offer or consent to follow-up does not result in loss of access to any existing HTS services. The follow-up surveys and secondary data analysis are approved by the University of Witwatersrand Human Research Ethics Committee (ethics reference number 180405). The design and evaluation of the self-report platforms are approved by the University of Witwatersrand Human Research Ethics Committee ethics reference number 171113, and 180708 for IVRS and PWA and WhatsApp for business respectively.

The dissemination plan for the study findings will include presentations to local, and international authorities at the NDOH, WHO, and at local and international conferences. In addition, the findings will be submitted for publications to open access journals with impact factor of >1.5 for wider visibility. To ensure that the findings help inform implementation of HIVST in areas most affected by the COVID-19 pandemic where HTS are disrupted, the findings will also

For peer review only

be shared with community-based organizations and practitioners involved in HTS at the community level in case they do not access.

For peer review only

References

1. National Strategic Plan. South African National Strategic Plan on HIV, TB and STIs 2017-2022 2017 [Available from: http://sanac.org.za/2017/10/24/download-the-full-version-of-the-national-strategic-plan-for-hiv-tb-and-stis-2017-2022/ accessed 18 Nov 2017.
2. Jiang H, Zhou Y, Tang W. Maintaining HIV care during the COVID-19 pandemic. *The Lancet HIV* 2020;7(5):e308-e09.
3. Ponticiello M, Mwanga-Amumpaire J, Tushemereirwe P, et al. “Everything is a Mess”: How COVID-19 is Impacting Engagement with HIV Testing Services in Rural Southwestern Uganda. *AIDS and Behavior* 2020:1.
4. WHO. Guidelines On HIV Self-Testing and Partner Notification - Supplement To Consolidated Guidelines On HIV Testing Services, 2016.
5. Mhango M, Chitungo I, Dzinamarira T. COVID-19 Lockdowns: Impact on Facility-Based HIV Testing and the Case for the Scaling Up of Home-Based Testing Services in Sub-Saharan Africa. *AIDS and Behavior* 2020:1.
6. Dovel K, Shaba F, Offorjebe OA, et al. Effect of facility-based HIV self-testing on uptake of testing among outpatients in Malawi: a cluster-randomised trial. *The Lancet Global Health* 2020;8(2):e276-e87.
7. Organization WH. WHO recommends HIV self-testing: evidence update and considerations for success: policy brief. WHO recommends HIV self-testing: evidence update and considerations for success: policy brief2019.
8. HIV Self-Testing AfRica (STAR) Initiative - Research London school of Hygiene and Tropical Medicine; 2018 [Available from: http://hivstar.lshtm.ac.uk/.
9. UNAIDS. Global AIDS update 2019—communities at the centre: UNAIDS Geneva, 2019.
10. Ingold H, Mwerinde O, Ross AL, et al. The Self-Testing AfRica (STAR) Initiative: accelerating global access and scale-up of HIV self-testing. *Journal of the International AIDS Society* 2019;22:e25249.
11. d’Elbée M, Indravudh PP, Mwenge L, et al. Preferences for linkage to HIV care services following a reactive self-test: discrete choice experiments in Malawi and Zambia. *Aids* 2018;32(14):2043-49.
12. Indravudh PP, Sibanda EL, d’Elbée M, et al. ‘I will choose when to test, where I want to test’: investigating young people's preferences for HIV self-testing in Malawi and Zimbabwe. *Aids* 2017;31:S203-S12.
13. Indravudh PP, Choko AT, Corbett EL. Scaling up HIV self-testing in sub-Saharan Africa: a review of technology, policy and evidence. *Curr Opin Infect Dis* 2018;31(1):14-24. doi: 10.1097/QCO.0000000000000426
14. UNITAID. World’s largest HIV Self-Testing Initiative expands in critical new phase. : World Health Organisation (UNITAID), 2018.
15. Neuman M, Indravudh P, Chilongosi R, et al. The effectiveness and cost-effectiveness of community-based lay distribution of HIV self-tests in increasing uptake of HIV testing among adults in rural Malawi and rural and peri-urban Zambia: protocol for STAR (self-testing for Africa) cluster randomized evaluations. *BMC public health* 2018;18(1):1234.
16. Shapiro AE, van Heerden A, Krows M, et al. An implementation study of oral and blood-based HIV self-testing and linkage to care among men in rural and peri-urban KwaZulu-Natal, South Africa. *Journal of the International AIDS Society* 2020;23:e25514.
17. Lagat H, Sharma M, Kariithi E, et al. Impact of the COVID-19 Pandemic on HIV Testing and Assisted Partner Notification Services, Western Kenya. *AIDS and Behavior* 2020:1.

For peer review only

For peer review only

Author's contributions

MM, DFC and VZ wrote the first draft of the manuscript. MM and FV designed the program implementation strategies, the operational study design. MM, AT, VZ oversaw the implementation of the program and drafted the standard operating procedures. JP oversaw data management processes. CJ and KH provided an oversight of the program implementation. DFC and ZTH helped to revise the manuscript and address major reviewers' comments. All authors took part in the revision, reading, and approved the final version of the protocol manuscript.

Funding

This work was funded by the UNITAID grant 2017-17-SFH-STAR and Bill & Melinda Gates Foundation OPP1132929, the National Institute of Health (R00MH110343: PI, DFC) and the HIV Dissemination Science Training Program for Underrepresented Investigators grant (R25MH080665). The findings and conclusions in this report are those of the authors and do not necessarily represent the views of the funder.

Competing interests

Authors have no competing interests.

For peer review only

Table 1: HIVST distribution models implemented by WitsRHI / Ezintsha

Model	Modality	Description
**Community Based Distribution**	Hotspots	HIVSS was offered to high-risk populations at hotspots through fixed pop-up sites. Hotspots include high foot traffic areas such as busy walkways or shopping centers. After a peer educator provided a demonstration on how to use HIVSS kits, clients were provided with a choice to take a kit home or use it onsite either on their own or with the assistance of the distributor. Clients opting to test onsite were given an opportunity to confirm reactive results. All women and males who indicated having a male partner were offered an HIVSS test kit for their partners. 5% of consenting primary recipients of HIVSS were telephonically followed up for outcomes of HIVSS use, result, linkage to care and uptake of ART. From November 2019, all consenting clients were offered a choice to self-report their outcomes using one of the 3 m-health platforms, specifically, WebApp, WhatsApp or on the interactive Voice Response system (IVRS).
	Transport Hub	The description of this modality is the same as the Community based- Hotspots modality, with a specific focus on distribution in transport hubs including Taxi ranks and Train stations
	Door to Door	Within a specific mapped community or geographic location, peer educators and/or trained counsellors move from one household to another and offer HIVSS to eligible clients residing there. Clients were offered HIVSS with the option of either directly assisted (in presence of health care provider) or unassisted. Clients who self-report reactive results were offered the option to confirm their results onsite. All women and men who had male partners were offered oral HIVSS tests for their partners. To collect information on outcomes of HIVSS use, result, linkage to care and uptake of ART, Peer educators went back to the residences of all consenting clients
	Integrated HTS (mobile)	This modality involved the Integration of HIVSS into existing mobile HIV testing activities. Clients were offered a choice between HIVSS onsite or Rapid Diagnostic Testing (RDT) with a counsellor. HIVSS kits were distributed to individual clients after a demonstration of how to use them. Clients that opted for HIVSS, were only offered onsite testing with the option to confirm a reactive HIVSS result immediately. Clients who confirmed reactive were referred to nearby health facilities for ART initiation. To collect information on outcomes of linkage to care and uptake of ART, linkage officers checked ART registers of referral health facilities. Integration of HIVSS into mobile HTS services did not displace traditional blood based RDT uptake but provided a screening option increased yield and efficiency for triaging clients for further managed health care.

For peer review only - http://bmjopen.bmj.com/site/about/guidelines.xhtml

Model	Modality	Description
**Key Population Distribution**	Sex Worker	This modality was implemented alongside the WitsRHI sex worker program, which provides various health services to sex workers at static sex worker clinics and at outreach sites. Sex workers were offered up to 5 HIVSS kits to take for their network which would either be; their non-client sexual partners; their clients; other sex workers not accessing HIV testing services; or member of family or friend considered by the sex worker to be at high risk of HIV. This modality was intended to be a secondary only distribution modality; however, sex workers could use one kit for themselves if they needed to test themselves together with one of their recipients. All consenting sex workers were telephonically followed up to determine the usage, linkage to care and ART initiation status of their networks.
**Public Sector Facility Distribution**	Outpatient Department (OPD)	Within the hospital out-patient waiting areas, peer educators created awareness and provided information on both traditional RDT and HIVSS. Clients were then offered the choice between testing themselves onsite using either a blood or oral based HIVSS test inside a provided cubicle or have RDT with a counsellor. HIVSS Reactive clients were provided with the opportunity to confirm their results and be initiated on ART immediately. Offering HIVSS alongside traditional rapid testing in the out-patient departments not only provides clients with a choice but also a triage system whereby testers who screen non-reactive using HIVSS may exit, allowing counsellors and nurses offering rapid testing enough time to focus on likely reactive testers.
**Private Sector Facility Distribution**	GP Practice & Nurse-led clinics	Clients attending health services at GP practices or Nurse-led Unjani clinics were offered HIV testing using HIVSS during their consultation. After a demonstration was provided by the consulting clinician, clients are offered a private testing space within the facility to test themselves and were asked to self-report their result. Confirmatory testing and ART initiation were available onsite. All clients who self-report a reactive result were offered an HIVSS test to take home for their partners.
**Private Sector Distribution**	Pharmacy	Pharmacists and pharmacist assistants at private pharmacies offered HIVSS to adult clients, 17 and older seeking services that might suggest HIV risk, including emergency contraceptive, treatment for STI's, condoms, lubricants, sexual performance enhancers etc. After a demonstration is offered, clients can choose to either test onsite on their own or take the kit home. Clients who chose to test onsite were given an opportunity to self-report their result to the Pharmacist or pharmacist assistant. All who self-report a reactive result, were offered onsite confirmatory testing when available. All women and men who have male partners were offered oral HIVSS tests for their partners. clients were offered m-health platforms to self-report on use, result, and linkage to care.

For peer review only - http://bmjopen.bmj.com/site/about/guidelines.xhtml

Model	Modality	Description
	Workplace	This involved distribution of HIVSS in formal workplaces with a focus on mining, manufacturing, construction, petroleum, agriculture, and security sectors. Employees were offered an HIVSS test kit to take home. All female employees were offered an HIVSS kit for their male partners. A 5% sample of primary consenting recipients of HIVSS were telephonically followed up to obtain information on HIVSS usage, result, linkage to care and ART initiation. Beginning August 2019, clients were also offered m-health platforms to self-report on use, result, and linkage to care.
**Secondary Based Distribution**	Antenatal	After a demonstration and social harm assessment, all 1st visit antenatal attendees were offered HIVSS to take home to support testing their sexual partners. The HIVSS modality was not intended to replace facility based RDT testing but support testing of male partners. Women could take HIVSS kit for herself to support home disclosure. Consenting women were followed up by telephone or at next visit to acquire information on whether HIVSS had been used by partner(s) and for test result and linkage data.
	Index Testing	HIVSS is offered to newly diagnosed HIV positive clients (ART clinic) to facilitate partner notification. Telephonic follow up of HIV positive index to ascertain index partner uptake of HIVSS, linkage of positive sexual partner to confirmative testing and treatment initiation

For peer review only
